# An efficient, scalable, and adaptable framework for solving generic systems of level-set PDEs

**DOI:** 10.3389/fninf.2013.00035

**Published:** 2013-12-26

**Authors:** Kishore R. Mosaliganti, Arnaud Gelas, Sean G. Megason

**Affiliations:** Department of Systems Biology, Harvard Medical SchoolBoston, MA, USA

**Keywords:** level-set, software, design, implementation, Insight Toolkit

## Abstract

In the last decade, level-set methods have been actively developed for applications in image registration, segmentation, tracking, and reconstruction. However, the development of a wide variety of level-set PDEs and their numerical discretization schemes, coupled with hybrid combinations of PDE terms, stopping criteria, and reinitialization strategies, has created a software logistics problem. In the absence of an integrative design, current toolkits support only specific types of level-set implementations which restrict future algorithm development since extensions require significant code duplication and effort. In the new NIH/NLM Insight Toolkit (ITK) v4 architecture, we implemented a level-set software design that is flexible to different numerical (continuous, discrete, and sparse) and grid representations (point, mesh, and image-based). Given that a generic PDE is a summation of different terms, we used a set of linked containers to which level-set terms can be added or deleted at any point in the evolution process. This container-based approach allows the user to explore and customize terms in the level-set equation at compile-time in a flexible manner. The framework is optimized so that repeated computations of common intensity functions (e.g., gradient and Hessians) across multiple terms is eliminated. The framework further enables the evolution of multiple level-sets for multi-object segmentation and processing of large datasets. For doing so, we restrict level-set domains to subsets of the image domain and use multithreading strategies to process groups of subdomains or level-set functions. Users can also select from a variety of reinitialization policies and stopping criteria. Finally, we developed a visualization framework that shows the evolution of a level-set in real-time to help guide algorithm development and parameter optimization. We demonstrate the power of our new framework using confocal microscopy images of cells in a developing zebrafish embryo.

## 1. Introduction

**Figure 1:**
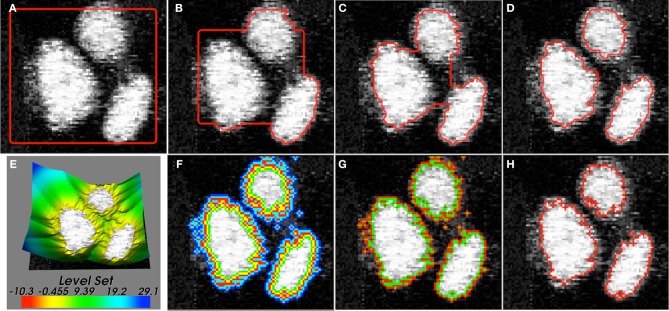
**(A–D)** Iterative stages of active contour (red) evolution using level-sets for the segmentation of objects (cells in this case) in images. The underlying image is a 2D confocal microscopy section showing nuclei (H2B:GFP) in the zebrafish inner ear. **(E)** The level-set function is typically defined over the entire 2D image domain. Different narrow-band representations are shown in **(F)** Whitaker, **(G)** Shi, and **(H)** Malcolm. The Whitaker method uses 5 layers, Shi uses 2, and Malcolm uses 1 layer.

The automated identification of anatomical structures found in medical and microscopy images is an important step in any imaging-based quantitative analysis pipeline. Large variations in image quality arising from differences in acquisition protocols, anisotropic point-spread functions, and image noise complicate the task of automated image analysis tools. To overcome the disadvantages associated with simple heuristic methods, a class of methods for contour evolution known as geometric active contour models, or level-sets, have been actively developed (Osher and Sethian, 1988; Sethian, 1999). Level-sets have become a preferred method for addressing a number of image science problems (Tsai and Osher, 2004) including denoising (Rudin et al., 1992; Dibos and Koepfler, 1998; Vese and Osher, 2004), registration (Vemuri et al., 2000, 2003; Droske and Ring, 2006), segmentation (Caselles et al., 1995; Malladi et al., 1995; Leventon et al., 2000; Cremers et al., 2006), tracking (Dufour et al., 2005; Dydenko et al., 2006; Dzyubachyk et al., 2010), and surface reconstruction (Zhao et al., 2001; Nilsson et al., 2005). Level-set methods represent the presumptive boundary 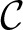 of an object of interest as the zero level-line of a higher dimensional implicit function 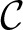
(*t*) = {(*x*, *y*)|ϕ(*x, y, t*) = 0}, also called the level-set function. For example, the boundary 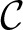 can be arbitrarily initialized along with an initial level-set function ϕ_0_(*x, y*) constructed as a signed distance function to 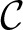. In Figure 1A, we plot the zero curve of a level-set function initialized with a square. Then, the evolution of the level-set function to match the true object boundary is governed by setting speed functions or via the minimization of energy functionals (Figures 1B–D). In a basic formulation, the evolution equation of the level-set function can be specified as follows:

(1) $$\begin{array}{l} \frac{\partial \phi }{\partial t}+F|\nabla \phi |=0\\ \;\phi (x,y,0)=\phi_{0}(x,\;y) \end{array}$$

The function *F* is called the speed function and depends on the image data *I* as well as ϕ. An advantage of the level-set method, especially for medical imagery, is its natural ability to incorporate information on object shape, texture, and color distribution into the segmentation process. Level-sets also avoid the problem of explicit parameterization of the object boundary, a problem with parametric active contour approaches such as snakes (Kass et al., 1988), thus providing flexibility in the segmentation of objects with topological changes, cusps, and corners. Furthermore, the same level-set formulation applied on 2D images naturally extends to an *N*-dimensional image. Given the large number of level-set methods already developed, we refer to (Osher and Fedkiw, 2004) for a complete exposition of the level-set calculus. We next discuss the major classes of level-sets methods and how they impact the development of a generic software system.

## 2. Background

In general, there are two main classes of the level-set methodology that arise from using a variational approach that minimizes an energy functional: (i) edge-based and (ii) region-based methods.

### 2.1. Edge-based level-sets

The goal of edge-based approaches is to evolve the contour until it finds an object edge where its speed is gradually reduced to 0. An edge-based level-set based on mean curvature motion is given by (Caselles et al., 1993):

(2) $$\begin{array}{l} \;\frac{\partial \phi }{\partial t}=g(\nabla I)|\nabla \phi |\left(div\left(\frac{\nabla \phi }{\left|\nabla \phi \right|}\right)+\nu \right)\\ \phi (x,y,0)=\phi_{0}(x,\;y) \end{array}$$

where ν ≥ 0 is a constant. The update equation is constructed from the image intensity (*I*), gradient (∇*I*), and edge function *g*(∇*I*). The edge function *g*(∇*I*) is designed to have values close to 0 at the boundary and large values elsewhere. The zero level-curve of this level-set moves with a speed of $g(I)|\nabla \phi |\left(div\left(\frac{\nabla \phi }{\left|\nabla \phi \right|}\right)+\nu \right)$ and stops on the object boundary where *g* approaches zero. The constant term ensures the level-set always advances toward the object boundary even when the curvature ($div\left(\frac{\nabla \phi }{\left|\nabla \phi \right|}\right)$) becomes negative, i.e., $\left(div\left(\frac{\nabla \phi }{\left|\nabla \phi \right|}\right)+\nu \right)$ remains positive. Another popular example of an edge-based level-set uses the strong image gradient at the object boundary to slow down or stop the zero curve (Malladi et al., 1993, 1994):

(3) $$\begin{array}{l} \;\frac{\partial \phi }{\partial t}=|\nabla \phi |\left(-\nu +\frac{\nu }{\left(M_{1}-M_{2}\right)}\right)\left(|\nabla G*I|-M_{2}\right)\\ \phi (x,y,0)=\phi_{0}(x,y) \end{array}$$

where ν is a constant, *G* is a Gaussian function, and *M*_1_ and *M*_2_ are the maximum and minimum values of the image gradient (|∇G*I|). In these level-sets, parts of the contour reach the boundary and cross-over before the rest of the contour catches up. In order to prevent the contour from over-shooting the edge so that it remains trapped in a minimum along the boundary, Kichenassamy et al. (1995) and Caselles et al. (1995) independently proposed the geodesic active contour formulation. Here, the underlying energy is representative of the contour length in a Riemannian space with a metric induced by the image intensity:

(4) $$\begin{array}{l} \;\frac{\partial \phi }{\partial t}=|\nabla \phi |div\left(g(I)\frac{\nabla \phi }{\left|\nabla \phi \right|}\right)+\nu g(|\nabla I|)\\ \;=g(I)|\nabla \phi |div\left(\frac{\nabla \phi }{\left|\nabla \phi \right|}\right)+\nabla g(I)\cdot \nabla \phi +\nu g(|\nabla I|)\\ \phi (x,y,0)=\phi_{0}(x,y) \end{array}$$

The first (curvature) and last (propagation) terms in Equation 4 approach zero at the object boundary. The middle (advection) term ensures that contour motion is always directed toward the boundary.

Many additional variants of edge-based concepts have been published. An abstract representation common to all edge-based partial differential equation (PDE) is as follows:

(5) $$\begin{array}{l} \;\frac{\partial \phi }{\partial t}=-\alpha A(x)\cdot \nabla \phi -\beta P(x)|\nabla \phi |+\gamma Z(x)\kappa |\nabla \phi |\\ \phi (x,y,0)=\phi_{0}(x,y) \end{array}$$

where *A* is an advection term, *P* is a propagation (expansion) term, and *Z* is a spatial modifier term for the mean curvature κ. The scalar constants α, β, and γ weight the relative influence of each of the terms on the movement of the interface. Based on this prevalent model in the early 2000s, level-sets were implemented in the NIH/NLM Insight Toolkit (ITK) v3. As we show next, this model is inadequate in its representation of region-based level-sets.

### 2.2. Region-based level-sets

Region-based level-sets segment the image into objects based on region statistics (rather than just object edges) of intensity, texture, or color values. For example, the region mean intensity for foreground (*c*_1_) and background (*c*_2_) is a popularly used statistic for defining an energy functional *F* whose minimization leads to an optimal segmentation of the foreground (Chan and Vese, 1999, 2001):

(6) $$\begin{array}{l} F(c_{1},c_{2},\phi )=\int_{Inside(C)}{\left(I(x)-c_{1}\right)}^{2}dx\\ \;+\int_{Outside(C)}{\left(I(x)-c_{2}\right)}^{2}dx+\nu \cdot Area(C)\\ \;+\mu \cdot Length(C) \end{array}$$

Minimizing the energy functional using the standard gradient descent (or steepest descent) method leads to the following gradient-flow equation:

(7) $$\begin{array}{l} \;\frac{\partial \phi }{\partial t}=\delta_{e}(\phi )[\mu .div\left(\frac{\nabla \phi }{\left|\nabla \phi \right|}\right)-\nu -\lambda_{1}{\left(I-c_{1}\right)}^{2}\\ \;+\lambda_{2}{\left(I-c_{2}\right)}^{2}]\\ \;c_{1}=\frac{\int_{\Omega }I(x,y).H(\phi (x,y))dxdy}{\int_{\Omega }H(\phi (x,y))dxdy}\\ \;c_{2}=\frac{\int_{\Omega }I(x,y).(1-H(\phi (x,y)))dxdy}{\int_{\Omega }\left(1-H(\phi (x,y))\right)dxdy}\\ \phi (x,y,0)=\phi_{0}(x,y) \end{array}$$

where *H* is the Heaviside function, δ_*e*_ is the smoothed Dirac-Delta function, and μ and ν regularize the curve length and foreground area, respectively. To account for image inhomogeneities and large gradients that may be present in an image, piecewise-smooth extensions were proposed in Tsai et al. (2001); Vese and Chan (2002); Li et al. (2008). In these extensions, local intensity-functions are defined in place of using constants *c*_1_ and *c*_2_ and the energy functional was defined in terms of the fit with respect to these functions. Nevertheless, the final equation form is similar to Equation 7.

Within the last decade, region-based techniques, as well as graph-partitioning-based active contours (Sumengen et al., 2004; Sumengen and Manjunath, 2006), have emerged. These new methods do not ascribe to the same generic PDE (Equation 5) used in ITKv3 framework. The addition of region-based methods in the ITKv3 framework required us to duplicate significant amounts of code (Mosaliganti et al., 2009a,b,c). Although edge and region-based methodologies arise from different strategies, our new software design and implementation in the ITK v4 combines the two hierarchies seamlessly with minimal code duplication. New terms can also be easily added and require no changes in the evolution and update classes.

### 2.3. Narrow-band methods

One of the primary disadvantages with using level-set methods for image segmentation is that they are slow and memory-intensive. The level-set function is typically discretized on the entire image grid to hold floating-point values (Figure 1E and Movie_Dense_S1.mov) although only the position of the zero level-curve is of primary interest. Therefore, a key development in reducing computational cost has been the emergence of narrow-band algorithms (Whitaker, 1998; Malcolm et al., 2008; Shi and Karl, 2008). These methods evolve the level-set function in a layer around the zero level-set (sparse-field) alone as opposed to its solution on the entire image domain (dense). While the Whitaker method (Whitaker, 1998) (Figure 1F and Movie_Whitaker_S2.mov) uses 5 layers around the zero level-curve, the Shi and Karl (2008) (Figure 1G and Movie_Shi_S3.mov) and Malcolm et al. (2008) (Figure 1H and Movie_Malcolm_S4.mov) implementations use 2 and 1, respectively. Together, they provide a significantly faster and less memory-intensive implementation although the trade-off is that the solution is only maintained around the zero level-curve. They may also lead to a different local solution closer to the initialization contour compared to the dense case. In current software systems, narrow-band implementations derive from the dense case and thus use an image-based representation and do not fully exploit the speed and memory enhancement possible. In our new software design, we derive the sparse level-set formulations in a separate hierarchy that represents the level-set function as linearized lists of pixels with floating-point values. The pixels map onto a label image stored in run-length encoded format. By introducing suitable narrow-band base classes, we have allowed for future enhancements and new narrow-band representations to be introduced without much effort or code-duplication in the ITKv4 framework.

### 2.4. Reinitialization and stopping criterion

During the evolution of the level-set function, the differential movement of different level-curves can cause the function to develop gradients that are too steep and/or too flat. This negatively impacts the numerical stability in subsequent iterations leading to unpredictable outcomes. Therefore, one needs to perform a distance reinitialization step at the end of every few iterations, such that the zero level-curve location remains unchanged. One way to perform such an initialization is to evolve the PDE to a steady-state according to Sussman et al. (1994):

(8) $$\begin{array}{l} \frac{\partial \phi }{\partial t}+sign(\phi_{0})(|\nabla \phi |-1)=0\\ \;\phi (x,y,0)=\phi_{0}(x,y) \end{array}$$

where ϕ_0_ represents the level-set function before the reinitialization. The end result will be a signed distance function to the interface (ϕ_0_ = 0). Another approach is to solve the Eikonal equation using fast-marching methods (Peng et al., 1999):

(9) $$\begin{array}{l} \;|\nabla \phi |=1\\ \phi (x,y,t)=0\;\Leftrightarrow \phi_{0}(x,y)=0 \end{array}$$

In this method, the signed distance function is used to fix the level-set function in a narrow band around the zero curve as boundary conditions. Fast marching methods are then used to solve the Eikonal equation.

In another development, Li *et al*. defined a new term in the equation that automatically maintains the level-set function to a signed distance function (Li et al., 2005).

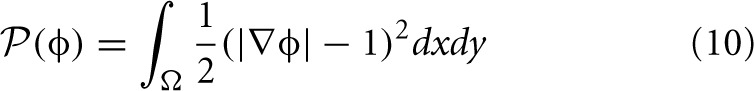



The addition of this energy followed by the steepest descent flow leads to the addition of a new term $(\Delta \phi -div\left(\frac{\nabla \phi }{\left|\nabla \phi \right|}\right))=div\left[(1-\frac{1}{\left|\nabla \phi \right|})\nabla \phi \right]$. In regions where |∇ϕ| > 1, this term causes an outward diffusion of level-curves. Similarly, where |∇ϕ| < 1, there is an inward diffusion of level-curves. The term approaches zero when |∇ϕ| = 1 leading to a solution of the Eikonal equation.

Similar to the reinitialization problem, the stopping criterion also presents several choices to the user. The evolution of a level-set function is typically halted by a threshold set on the number of iterations (*N*), and/or by assessing the reduction in the variational energy, and/or by assessing the change in the level-set function, and/or by checking to see if the level-set has reached certain pre-set boundary points. In order to let users explore the above strategies and possibly develop new ones, we implemented reinitialization and stopping base classes that serve as plugins into the level-set evolution framework in ITK v4. This provides complete flexibility to the user in developing a customized implementation of level-sets without restrictions from the design.

### 2.5. Multi-object and multiphase methods

In biomedical and biological image analysis applications, one is often interested in segmenting more than a single object (of the same or different kind) from a given image. This especially happens when the objects to be segmented are adjacent to each other and the delineation of one object automatically affects the neighboring object. For example, in MRI imagery, white and gray matter regions in the brain have a common interface. Moreover, spatial constraints often specify relationships between classes of level-sets. For example, microscopy images depict thousands of cells in terms of their plasma membranes, nuclear membranes, organelles, and proteins. Here, the nucleus and organelles are always contained within the membrane. In such situations, concurrently interacting and evolving level-sets provide optimal and efficient image segmentation solutions. Multi-object methods (Dufour et al., 2005; Mosaliganti et al., 2009a,c) use a unique level-set function per anatomical structure in the image. Overlaps among the level-set functions can be penalized or encouraged through a special term placed in each of the level-set update equations. Another strategy is to use multiphase methods, where different phases of the set of level-set functions (+ and − parts) encode a unique anatomical structure (Vese and Chan, 2002). While multiphase strategies work well for a small number of objects, multi-object strategies are more suitable for the case of a larger number of objects. In our new software framework, we enable the solution for a large system of level-set equations. No restriction is placed on the type of level-sets (dense or sparse). Each level-set function may evolve according to a different update equation specified by the user. Furthermore, the number of level-set functions in the system may dynamically change during iterations (say, from cell division or from a cell entering or exiting the field-of-view during tracking). The individual terms of the update equations can also dynamically change. Thus, accounting for such flexibility in the design allows one to fully exploit the level-set techniques.

## 3. Methods

**Figure 2:**
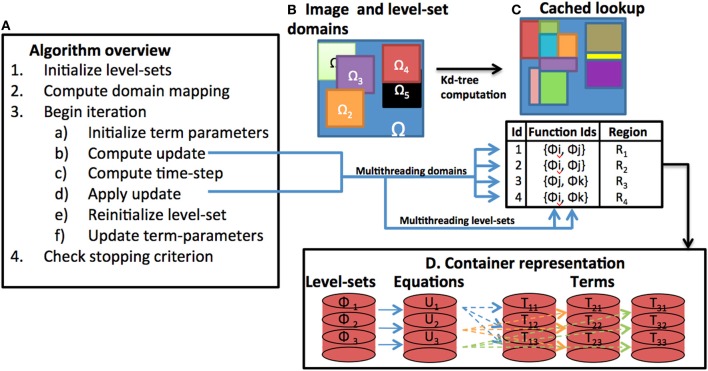
**Components of the level-set framework. (A)** An overview of the basic level-set algorithm is provided. **(B)** Individual level-set domains are assumed to be subsets of the image domain. **(C)** The domain is partitioned into a label-map that specifies the interacting level-set ids and the corresponding subregion. **(D)** Level-set functions, equations, and, terms are stored in a container format. During iteration, the computation of the level-set updates are multithreaded either in terms of processing individual domains or individual level-set functions.

**Figure 3:**
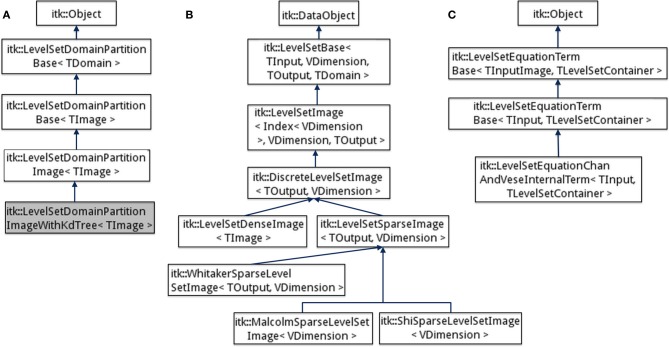
**Inheritance diagrams for (A) Domain partitioning classes, (B) Level-set function, and (C) Level-set equation term classes in the ITK v4 framework**.

The goal of the new inheritance framework is to permit the implementation of a variety of level-set methods with minimal code-duplication and allow end-users to explore and customize the method to their data. The new framework is designed to be modular, and therefore, reduces the effort needed to expand the level-set code base. An overview of the level-set algorithm with components of our framework is provided in Figure 2. Corresponding class inheritance diagrams for the components are shown in Figures 3, 5. We elaborate on the components of the new framework next.

### 3.1. Domain representation

While the level-set function has typically been implemented on underlying image grids, it also theoretically extends to unstructured grids or surface meshes and continuous representations. In the popular ITK v3 framework, for example, an image representation was used throughout the code which impedes the development of mesh-based level-set segmentation methods. Additionally, in a continuous representation, the level-set function is not discretized on a grid but instead represented analytically in terms of linear combination of base functions [e.g., radial basis functions (Bernard et al., 2007; Gelas et al., 2007), splines (Bernard et al., 2009)]. Such an underlying representation may still use the same energy formulation and equation terms. Thus, modularizing the level-set code base such that the term and equation classes do not directly depend on the domain representation is essential for building a common framework. We next detail how the level-set function is defined to account for all such representations.

### 3.2. Level-set function

Our code is heavily templated to allow for a great deal of flexibility in level-set methods in an efficient manner. In our new framework, we define an abstract level-set function base class (itk::LevelSetBase) inheriting from itk::DataObject based on 4 template parameters (Figure 3A).


template< class TInput, unsigned int
VDimension, typename TOutput,
  class TDomain > class LevelSetBase :
public DataObject


where TInput defines the input type where the level-set function will be evaluated, VDimension is dimension of the input space, TOutput is the returned type when evaluating the level-set function (for the general case when it is not a scalar), TDomain is the discretization of the level-set function (e.g., ImageBase or QuadEdgeMesh). In the case of an image representation, this specializes as only the first three parameters and inherits from the itk::ImageBase class:

template< class TInput, unsigned int
VDimension, class TOutput > class
LevelSetImage : public LevelSetBase<
TInput, VDimension, TOutput, ImageBase<
VDimension > >


While this definition accounts for an image representation (continuous or discrete), we further specialize into a discrete image representation with TInput as itk::Index to enable the traditional image-discretized implementation of the level-set method.


template< typename TOutput, unsigned int
VDimension >
class DiscreteLevelSetImage : public
LevelSetImage< Index< VDimension >,
VDimension, TOutput >


All of the above level-set function classes implement specific member functions for returning the level-set value [ϕ(*x, y*)], gradient (∇ϕ), Hessian (∇^2^ ϕ), Laplacian (ϕ_*xx*_ + ϕ_*yy*_), gradient norm (|∇ϕ|), and mean curvature ($\kappa =div(\frac{\nabla \phi }{\left|\nabla \phi \right|})$) given its underlying representation (continuous or discrete image or mesh). Thus, the level-set equation, term, and evolution classes are independent of the underlying domain representation which facilitates the implementation of a wide variety of level-set methods.

The level-set quantities are stored as specific instances of a templated class itk::LevelSetBase::DataType<T>. The template parameter T determines whether the stored member variable is a scalar, vector, or tensor. The class contains three member variables that store the quantity name, value, and a Boolean flag indicating if it had already been computed. The level-set quantities are then collectively instantiated inside a special structure called itk::LevelSetBase::LevelSetDataType. When computing the update in a level-set equation (Figure 2A), the different terms in the equation repetitively compute the level-set quantities. Therefore, by passing the LevelSetDataType object among various terms, we ensure that level-set quantities are computed exactly once, cached, and reused. This significantly reduces the computations involved for calculating each term and the update overall.

The discrete image representation itk::DiscreteLevelSetImage is then specialized into the dense (itk::LevelSetDenseImage) and sparse (itk::LevelSetSparseImage) cases, which in turn was specialized into three sparse representations (itk::WhitakerSparseLevelSetImage, itk::ShiSparseLevelSetImage, and itk::MalcolmSparseLevelSetImage) (Figures 1E–H, 3B). The sparse representation (also sometimes called as narrow-band) discretizes the level-set function only around the zero level-curve. The number of layers and their update schemes differ among the three variants. A layer is defined as a map data-structure that links image indices to level-set function values. Each layer is associated with a unique id and the set of all the layers are stored as another map data-structure linking the id values to the layer. Additionally, all the sparse-field classes store a label-map (run-length image encoding) of the layers which significantly reduces the amount of memory used over a regular image (Lehmann, 2007).

#### 3.2.1. Image to level-set adaptors

For the convenience of end-users, adaptors for converting from binary images to level-set function objects were developed in ITK v4. The supplied binary image could be the output of a preprocessing segmentation pipeline. We consider the dense level-set function as well as the three types of sparse-field representations: (1) Whitaker, (2) Shi, and (3) Malcolm. The adaptor base class is templated over the input image type and the output level-set function type. It contains member variables for the input image and the computed level-set function representation and a pure virtual member function for computing the level-set function.


template< class TInputImage, class
TLevelSet > class
  BinaryImageToLevelSetImageAdaptorBase :
public Object


The derived class itk::BinaryImageToLevelSetImageAdaptor uses the partial template specialization mechanism for defining the virtual member function corresponding to each level-set representations.

### 3.3. Restricted level-set domains

The large computational costs associated with level-set methods is a particularly severe problem in multi-level set implementations. Typically, depending on the computing environment, one cannot evolve more than tens of level-set functions without running into memory problems. To address this problem, our new framework handles level-set functions defined on a subset of the input image domain (Ω). The interaction among level-set functions is limited to those functions whose domains overlap (Figures 2B,C). A helper base class (LevelSetDomainPartitionBase) is used to define the location and size of the level-set domains relative to Ω (Figure 3A). The domain of each level-set function is stored in a vector (m_LevelSetRegionVector). The class contains two pure virtual methods AllocateListDomain() and PopulateListDomain() that allocate and populate a new data-structure (mesh or image) depending on the underlying grid. Each grid point stores a list of the active level-set function ids. The class is specialized to LevelSetDomainPartitionImage in the case of a discrete image grid and to LevelSetDomainPartitionMesh for the case of meshes. For the case when there are thousands of level-sets, populating a list image by checking overlap at each pixel is time-consuming. Therefore, we further specialized into a class itk::LevelSetDomainPartitionImageWithKdTree. This class uses a *K*d-tree data structure that contains the centroids of the level-set domains. The *K*d- tree is used to query nearby level-set functions at each pixel and check for overlap. This enables the simultaneous evolution of thousands of level-set functions thereby expanding the applicability of level-set procedures to tracking large numbers of objects and in large images. Note that there is an initial overhead associated with building the *K*d-tree that can be avoided for cases involving a small number of level-set functions.

The output of the helper class is to define an image of list pixel types. Each list holds the ids of level-set functions active at that pixel. By using another helper class (itk::LevelSetDomainMapImageFilter), the list image is further clustered into sub-regions based on pixel types. Each sub-region has the same set of active level-set ids and can therefore be iterated upon for computing the update efficiently (Figure 2C). The itk::LevelSetDomainMapImageFilter is a member of the itk::LevelSetContainerBase.

**Figure 4:**
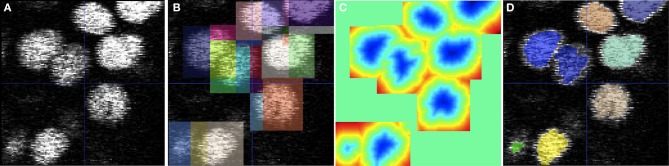
**Cell segmentation using multiple level-sets defined on subsets of the image domain. (A)** 2D confocal image of cell nuclei in the zebrafish ear. **(B)** Square domains defined around approximate centers of each cell. **(C)** Visualization of the overlapping level-set functions. **(D)** Final segmentation obtained using the geodesic active contour method. The level-set used advection, propagation, curvature, and overlap penalty terms with weights of 3, 3, 1, and 1000, respectively.

As an illustrative example,Figure 4 shows the application of the domain partitioning technique on a sample 2D confocal image of the zebrafish ear. In Figure 4A, we show closely-packed nuclei with varying intensities and noise. Figure 4B shows rectangular domains placed around each nuclei that define the extent of the initialized level-set function. The rectangular domains are subdivided to encode a unique level-set interaction within. Figure 4C shows the final level-set function and Figure 4D shows an overlay of the segmentation on the raw image. The small rectangular domain of each level-set does not influence its evolution and final segmentation but dramatically reduces the memory utilized and total running-time.

**Figure 5:**
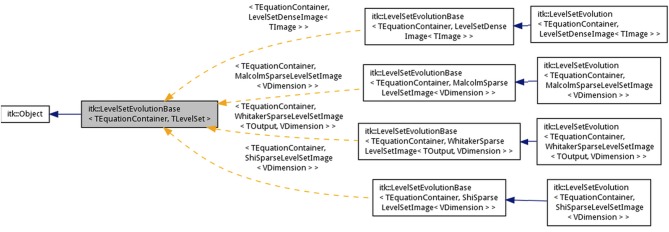
**Inheritance mechanism for level-set evolution classes**.

### 3.4. Terms

The level-set equation is a weighted sum of terms. Each term is a simple function of the level-set (e.g., gradient, Hessian, divergence, etc.), the input image (intensity, gradient, etc.), or involving the other level-set functions concurrently evolving in the system. As mentioned before, level-set computations occur repeatedly in each term and therefore values can be computed, cached, and stored in the level-set object and shared among the various terms. The term base class implements functions [Evaluate(.)] for computing the contribution from a term toward the level-set update. It also contains member functions for initializing/updating term parameters before [Initialize()]) and after [Update()] an iteration. While these functions are used for updating parameters in a dense level-set method, concurrent updating [UpdatePixel(.)] is carried out in a sparse level-set method. This is because only small incremental changes occur in the level-zero curve position in a sparse level-set method. Concurrent updating ensures that term parameters stay current and cuts down on computations in between iterations. This further enhances the speedup from using sparse level-set methods. The computed update is finally scaled by a weight data member (m_Coefficient). The term class also stores a level-set function identifier to supply the computed update to the evolution of the corresponding level-set function in the container. The term base class is templated using two parameters, namely, the input image type and a container of level-set functions (Figure 3C):

template< class TInputImage, class
TLevelSetContainer >
class LevelSetEquationTermBase : public
Object


Different types of terms arising from edge-based and region-based level-set methods such as the propagation, Laplacian, advection, curvature, and region-based terms described in Equations 4 and 7 derive directly from LevelSetEquationTermBase:

template< class TInput, class
TLevelSetContainer>
  class LevelSetEquationLaplacianTerm :
  public LevelSetEquationTermBase< TInput,
TLevelSetContainer >


### 3.5. Container-based design

Consider a concurrently evolving system of level-set functions {ϕ_1_, ϕ_2_, …, ϕ_*M*_} with input image *I*, and domain Ω. The generic level-set equation (*U_i_*) consists of a weighted summation of a number of different terms *T*_*ij*_.

$$\begin{array}{l} U_{1}:\frac{\partial \phi_{1}(p)}{\partial \tau }=\sum_{j=1}^{K_{1}}\alpha_{1j}\cdot T_{1j}(I,{\left\{\phi_{i}\right\}}_{i=1}^{M})\\ \;U_{2}:\frac{\partial \phi_{2}(p)}{\partial \tau }=\sum_{j=1}^{K_{2}}\alpha_{2j}\cdot T_{2j}(I,{\left\{\phi_{i}\right\}}_{i=1}^{M})\\ \;\vdots \\ U_{M}:\frac{\partial \phi_{M}(p)}{\partial \tau }=\sum_{j=1}^{K_{M}}\alpha_{Mj}\cdot T_{Mj}(I,{\left\{\phi_{i}\right\}}_{i=1}^{M}) \end{array}$$

The set of terms that constitute each equation may vary across all the *M* equations. Additionally, the number of concurrently evolving level-set functions in the system may dynamically change. To enable such flexibility, we used containers to store level-set function objects, equation objects, and their constitutive terms (Figure 2D). In each case, the container holds elements along with an id that is common across the level-set function, equation, and term containers. They are implemented as class templates, which allows a great flexibility in the types supported as elements. The container manages the storage space for its elements and provides member functions to access them, either directly or through iterators (reference objects with similar properties to pointers) or by using the id.

#### 3.5.1. Level-set container

In the case of multiple level-set functions, a special container class (itk::LevelSetContainerBase) was templated over two input parameters, namely, identifier type of the level-set function and the level function type (dense or sparse). The class provides iterators (const and non-const) and member functions for adding/removing individual level-set objects [AddLevelSet() and GetLevelSet()] by using the level-set identifier. Member functions also allow for copying and performing logical comparisons among container objects. A domain map filter (m_DomainMapFilter) is also instantiated that describes how the domain is split among the different level-set objects (see section 3.3).


template< class TIdentifier, class
TLevelSet >
  class LevelSetContainerBase : public
Object


Using the partial template specialization mechanism, the class itk::LevelSetContainer is specialized depending on the type of the level-set function. The derived class implements a member function for allocating new memory and copying individual level-set functions [CopyInformationAndAllocate(.)]. For example, the following class specializes the definition for the dense level-set method:

template< class TIdentifier, class TImage >
class LevelSetContainer< TIdentifier,
LevelSetDenseImage< TImage > > :
public LevelSetContainerBase< TIdentifier,
LevelSetDenseImage< TImage > >


#### 3.5.2. Term containers

For each level-set function defined by its identifier (CurrentLevelSetId), a term container class is instantiated that holds all the terms in its level-set equation. Using AddTerm() and PushTerm() member functions, new terms are introduced/removed from the level-set equation. While calculating the update to a level-set, the term container object is iterated and each term is evaluated and summed up to yield the update. Thus, member functions for initializing [InitializeParameters()], updating individual term parameters [Update()], and evaluating all terms [Evaluate()] are provided.


template< class TInputImage, class
TLevelSetContainer >
  class LevelSetEquationTermContainer :
public Object


The class is templated over the input image type and the level-set container type. The class is not derived further since there is no further specialization of the individual terms. In a given term container, the end-user can combine edge-based and region-based terms for customizing the level-set method which is yet another aspect of flexibility enabled by our framework.

#### 3.5.3. Level-set equation container

For a system of level-set equations, each equation (*U_i_*) corresponds to a unique term container. The class LevelSetEquationContainer is templated over the type of the term container. LevelSetEquationContainer holds the individual term containers for all the level-set functions. A unique term container can be referenced by the level-set function identifier (*i*) through GetEquation() member function. New equations can be added to the container using AddEquation(). While calculating the update to a level-set, the equation container object is iterated and each term container is in turn updated UpdateInternalEquationTerms(). Thus, member functions for initializing (InitializeParameters()) and updating individual term parameters (Update()) are provided.


template< class TTermContainer >
class LevelSetEquationContainer : public
Object


### 3.6. Level-set evolution

The class itk::LevelSetEvolutionBase implements the iterations in the level-set method in the Evolve() member function. At the beginning of each iteration, update buffers are allocated [AllocateUpdateBuffer()] and term parameters are initialized [InitializeIteration()]. Assuming the stopping criterion function is not satisfied, the iteration proceeds by computing the updates in the equation container, which in turn calls the individual term containers. The updates are computed by stepping through smaller domains where the active level-set function ids are known (from section 3.3). This avoids the costly step of checking at each pixel if a particular level-set is active. Based on the updates computed, the overall time-step for evolving the level-set is determined [ComputeTimeStepForNextIteration()]. Based on the time-step, the level-set functions are updated [UpdateLevelSets()] and the term parameters are then updated [UpdateEquations()]. Based on the change in the level-set functions, the global change is accumulated and set as input to the stopping criterion function. This function (described next) determines whether the next iteration continues. The base class is templated over the equation container type and the level-set function type.


template< class TEquationContainer, class
TLevelSet >
  class LevelSetEvolutionBase : public
Object


Dense and sparse-strategies are different in terms of how the update buffers are setup. Sparse methods visit only the narrow band layers while the dense method visits every pixel in the level-set domain. Therefore, as before we use partial template specialization to specialize the implementation depending on the level-set representation.

**Figure 6:**
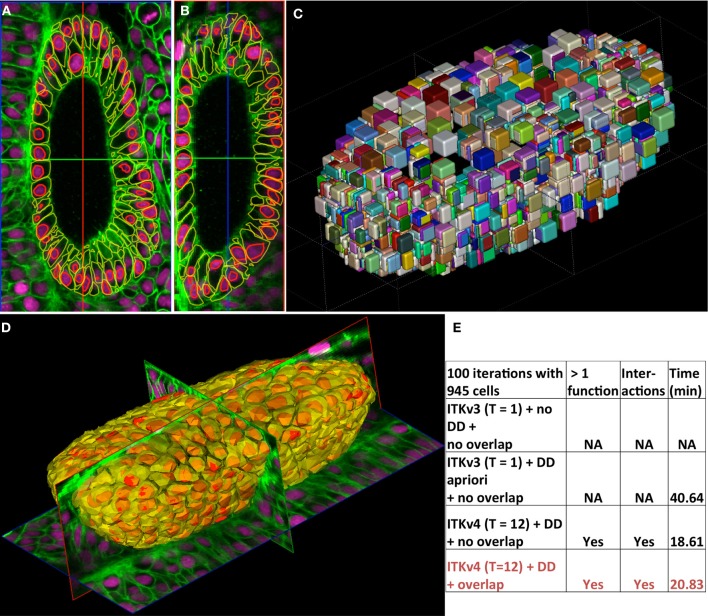
**Demonstration of cell segmentation in large 3D nuclear+membrane confocal microscopy data of a developing zebrafish ear at 24 h post-fertilization**. The images depict cell membranes in green (membrane-targetted mCitrine) and nuclei in magenta (H2B-tdTomato). The images are of dimensions 1024 × 1024 × 72 with a pixel sampling of 0.2 μm × 0.2 μm × 1.0 μm. A total of 485 whole cells and 461 nuclei were automatically identified by seeding level-sets at centers first identified by a Hough transform. **(A)** XY and **(B)** XZ sections showing membrane and nuclei segmentations in yellow and red contours, respectively. **(C)** A 3D randomly-colored rendering of the individual level-set domains. **(D)** A 3D rendering showing the segmentations all the ear cells with the tri-planar cross-sections of the image data. **(E)** Performance comparison between the old ITKv3 and the new ITKv4 frameworks, the number of threads used (T), with domain-discretization (DD), and accounting for level-set overlaps. The columns indicate if the framework can process multiple level-sets, if they allow interaction, and their running times, respectively. NA, not available

Figure 6 shows the application of our framework to the automated segmentation of cells from 3D confocal images of a developing zebrafish embryo expressing fluorescent proteins. The images show nuclei in magenta and membranes in green. The high-resolution images are of dimensions 1024 × 1024 × 72 with a pixel sampling of 0.2 μm × 0.2 μm × 1.0 μm. Using the Chan and Vese (1999, 2001) region-based terms with weights set to 1 (see section 2.2 and Equation 7) and an overlap penalty term of 1000, we evolved a total of 946 level-sets (485 cells and 461 nuclei) (Dufour et al., 2005). Level-sets were initialized as small spheres with a radius of 3.0 μm based on cell centers that were previous identified by using a Hough transform for identifying spherical objects. Figures 6A,B show the XY and XZ planes. Membrane and nuclei segmentations are contoured in yellow and red, respectively. Figure 6C shows the domain partitions from the nuclei level-sets alone. Figure 6D shows the a three-dimensional rendering of the segmentation after 100 iterations of the system.

The concurrent segmentation of all cells and corresponding cell nuclei in ITKv3 is intractable given that each level-set function would occupy the same domain as the image and 946 level-set functions would not fit in memory. Secondly, the level-sets would have to be segmented independently one function at a time. This would lead to a running time of many hours. Thirdly, level-set functions cannot interact with each other. Figure 6E highlights these differences between ITKv3 and the new framework in regards to the segmentation problem. In order to make meaningful comparisons, we performed domain partitioning in ITKv3 apriori by using region-of-interest filters outside the level-set API. While a single level-set is evolved at a time, several intermediate processing filters such as those involved in computing level-set reinitialization are intrinsically multithreaded. Thus, ITKv3 execution, is partially multithreaded.

In ITKv4, domain partitioning happens automatically and the user exactly specifies the number of threads to use. We provided running times for the procedure (≈21 min). *DD* refers to domain partitioning and *T* refers to the number of threads used. We also show running times without overlap (≈19 min) and with the ITKv3 framework (≈41 min). Thus, our method improves significantly over ITKv3 in speed of computation even after external domain discretization as well as ease of use and available options. Finally, we also evolve geometrically interacting level-sets that register a small increase in running time.

### 3.7. Stopping criterion

The iterations in the level-set method end when the functions finish evolving (converge) or when they satisfy some user-defined criterion. A specialized stopping criterion class (itk::StoppingCriterionBase) is implemented and checked at the end of each iteration [IsSatisfied()]. Typically, the method is terminated when there is no appreciable change in the level-set function or in the variational energy being minimized (itk::LevelSetEvolutionStoppingCriterion). In some applications, it is more useful to set a higher-limit on the iteration number (itk::LevelSetEvolutionNumberOfIterationsStoppingCriterion). In other applications, the arrival at a certain fraction of user-defined boundary points might be more meaningful. Thus, the new design enables many such approaches by allowing users to choose the appropriate stopping criteria.

### 3.8. User-interaction

In order to enable communication among filters and with graphical user-interfaces (GUIs), we used the itk::EventObject classes. These provide a standard coding interface for sending and receiving messages indicating the initiation/termination of processes and modification of filters. At the end of each iteration of the level-set method, an event [IterationEvent()] is triggered. This allows users to use observers (callbacks) on the itk::LevelSetEvolution object to execute a command [AddObserver(const EventObject & event, Command ^*^)]. Examples of downstream operations may include updating a progress bar in a visualization pipeline or in an interactive level-set method where the user can seed or terminate level-sets. This user-interaction implementation follows the subject/observer design pattern.

### 3.9. Visualization

To enable the real-time visualization and recording of level-set function evolution, we provide a pipeline linking our level-set framework to The Visualization Toolkit (VTK). The process of visualization consists of first mapping a level-set function to a VTK dataset. The base class for converting a itk::LevelSetFunction object is derived from itk::ProcessObject and templated over the level-set function type.


template< class TLevelSet >
class LevelSetToVTKImageDataBase : public
ProcessObject


Given the dense and three sparse-field representations of level-set functions implemented (Figures 1E–H), we specialize a class itk::LevelSetToVTKImageData for each type. In the dense level-set, the underlying level-set function is sampled on the image grid and passed to a VTK image defined on the same grid. In the sparse cases, the label-maps storing the locations of the sparse layers are converted into an ITK image and then to a VTK image. Having mapped the level-set function to a VTK image, the second step consists of developing suitable visualization of the level-set function using an image actor, renderer, and a rendering window that is annotated with the current iteration number. For a dense level-set function, one way is to visualize the level-set function with a simple colormap (itk::VisualizeImageLevelSet). Another option, only available in a two-dimensional case, is to visualize the level-set function as a elevation map by using a surface mesh representation (itk::VTKVisualize2DLevelSetAsElevationMap) (Figure 1E). At each point (*x, y*) in the grid, the level-set function is evaluated [*z* = ϕ(*x, y*)] to build a set of interconnected vertices. The mesh is color-mapped based on the height (*z*). A third option is to visualize the level-curves (user-defined isovalues) of the function (itk::VTKVisualizeImageLevelSetIsoValues) (Figure 1D). For this, the marching squares (in 2D), marching cubes (in 3D) algorithm are used to extract contours/meshes from the image and then color-mapped. For the sparse representations, visualization consists of rendering the layers around the zero curve of the function using a colormap (itk::vtkVisualize2DSparse-LevelSetLayersBase). Each layer is mapped to a unique user-defined color (Figures 1F–H). Like before, the class is specialized using partial template specialization for the three sparse representations because they contain different numbers of layers. Evolution of level-sets using the dense (Supplementary Movie S1) and the three narrow-band representations (Supplementary Movies S2-S4) can be visualized in real-time using the interaction classes described in Section 3.8 and the visualization classes described above. The level-sets were initialized as a square and segment the cell nuclei image shown in Figure 1 for 100 iterations.

## 4. Implementation and discussion

We chose to implement our framework using C++ in the NIH/NLM Insight Toolkit (ITK) v4 because of its solid software engineering practices, permissive license, community-support, and its popularity in the biomedical imaging community. The level-set classes, examples, and associated tests have been integrated into the ITKv4 toolkit. Detailed instructions for downloading ITK are available at: http://www.itk.org/Wiki/ITK/Git. Instructions for compiling and installing ITK on all common computer systems are available at: http://www.itk.org/ITK/help/documentation.html.

After compiling and installing ITK, users can navigate to the downloaded source directory and find a total of 123 level-set classes at the following location: ITK/Modules/Segmentation/LevelSetsv4/include. The level-set visualization classes that work with the Visualization Toolkit (VTK) are located at: ITK/Modules/Segmentation/LevelSetsv4Visualization/include. Documentation for the level-set classes detailing the member functions, variables, and inheritance hierarchy is available online at: http://www.itk.org/Doxygen44/html/group__ITKLevelSetsv4.html Each of the level-set classes is subject to unit tests individually as well as integrative tests. A total of 67 tests using simple datasets have been created to test the framework automatically. The tests are located at: ITK/Modules/Segmentation/LevelSetsv4/test. The tests are automatically compiled when building ITK and also provide further examples of code usage and API to new users. Users can run the tests from the compiled binary directory. The results of the automatic testing are reported on hundreds of computers across the world on a daily basis at: http://open.cdash.org/index.php?project=Insight. The results indicate whether the tests successfully configured, compiled, and executed on the remote system and also provide running times.

Additional examples of the level-set API used to generate the results for this article (Figure 1 and Supplementary Movies S1–S4) have been provided online in the ITKExamples repository: https://github.com/InsightSoftwareConsortium/ITKExamples. The repository includes 2D test image data and information for compiling and running our code. Other examples of ITK APIs and level-set code are also available online in a third repository at:http://www.itk.org/Wiki/ITK/Examples#ImageSegmentation. Finally, as open-source developers, we provide continuous feedback and troubleshoot problems reported by users on the ITK mailing lists:http://www.itk.org/ITK/help/mailing.html.

Thus, our framework addresses a long-standing challenge in the image analysis community for developing a state-of-the art ITKv4 segmentation and analysis toolkit. Our framework achieves the following goals:

### 4.1. Scalability to large datasets and extensibility to new methods

Current medical and microscopic imaging modalities feature rich datasets that requires the continuous development of new algorithms and performance optimization strategies to handle large data. In our framework, we combined the two major classes of edge-based and region-based methods seamlessly which should promote the development and usage of hybrid methods. We refactored sparse-field implementations to enable the addition of Whitaker, Malcolm, and Shi methods for improving performance. We enabled the implementation of multi level-set methods to handle multiple objects in images and also implemented domain partitioning techniques for improved performance. Thus, our framework supports large datasets, incorporates all the major level-set technologies, and enables the research and development of new ideas in the near future.

### 4.2. Adaptability to user-customizations and dataset variations

Our implementation allows the user to customize all aspects of the level-set method. The user can optimize the number of level-sets to use and specify how they interact. Users can specify the exact terms in the level-set update equations and choose the reinitialization and stopping criteria to use after each iteration. The large number of customization options available means that the user is better equipped to handle the varying challenges presented by different biomedical datasets. Users can also seed or terminate level-set functions, or manipulate the terms and term weights at the beginning of each iteration in the system. This paves the way for the exploration and development of real-time level-set systems in the future.

### 4.3. Efficient performance by multithreading, sparse-field, and domain partitioning strategies

The three sparse-field strategies significantly improve level-set performance on large datasets. Domain partitioning significantly reduces the memory utilized per level-set especially when the objects occupy a small portion of the large input space. By caching and reusing level-set quantities in the term calculations (see Section 3.2), we further improve performance. We also implemented multithreaded execution of the iterations to take advantage of multicore processor systems. Multithreading is accomplished by allocating threads to iterate on (i) different regions in the domain partitioned image or (ii) across individual level-sets. This choice is decided at run-time depending on the maximum number of threads available to the system, the number of level-sets initialized by the user, and the number of domain partitions present.

## 5. Future work

Currently, there is no other comprehensive framework or toolkit similar to the ITK framework in terms of implementing a multitude of level-set technologies in a scalable and efficient manner. However, upon further testing we do find that ITKv3 performs 2–2.5× faster for very basic segmentation using just one thread. This speed difference appears to result from the use of image iterators in ITKv3 compared with GetPixel() and SetPixel() methods in ITKv4 so future work could see a speed improvement by transitioning to image iterators. In the future, we plan to develop user-friendly APIs for developers familiar with ITKv3 and GUIs that will benefit new users. For the purpose of cell segmentation in microscopy, we have already begun developing a image analysis GUI called GoFigure2 (www.gofigure2.org). Currently, level-set implementations and applications have been restricted to discretized image domains. However, the level-set calculus is quite generic and can be extended to unstructured grids like meshes and analytically to continuous representations. Mesh segmentations are useful, for example, to segment surfaces. In the microscopy domain, this could serve to identify gene expression patterns on fly embryo surfaces or morphogen gradients on the tissue surfaces. Continuous representations can potentially give rise to faster level-sets on high-resolution image datasets. Real-time segmentation and tracking, and human-computer interactive applications are another major problem domain that could benefit from level-set appproaches. Real-time manipulation of level-sets in terms of the number of functions, their individual terms, and interactions can now be accomplished by our adaptive design.

## Author contributions

Arnaud Gelas designed the software. Kishore R. Mosaliganti and Arnaud Gelas implemented and tested the level-set software in C++. Arnaud Gelas developed the level-set visualization code. Kishore R. Mosaliganti and Arnaud Gelas ported the code into the official NIH ITK repository and continue to provide technical support. Kishore R. Mosaliganti analyzed the microscopy data reported in the article. Kishore R. Mosaliganti and Sean G. Megason wrote the manuscript. All authors discussed the manuscript and contributed to writing.

### Conflict of interest statement

The authors declare that the research was conducted in the absence of any commercial or financial relationships that could be construed as a potential conflict of interest.
